# Unconscious classification of quantitative electroencephalogram features from propofol versus propofol combined with etomidate anesthesia using one-dimensional convolutional neural network

**DOI:** 10.3389/fmed.2024.1447951

**Published:** 2024-09-18

**Authors:** Pan Zhou, Haixia Deng, Jie Zeng, Haosong Ran, Cong Yu

**Affiliations:** ^1^Department of Anesthesiology, Stomatological Hospital of Chongqing Medical University, Chongqing, China; ^2^Chongqing Key Laboratory of Oral Diseases and Biomedical Sciences, Chongqing, China; ^3^Chongqing Municipal Key Laboratory of Oral Biomedical Engineering of Higher Education, Chongqing, China; ^4^College of Artificial Intelligent, Chongqing University of Technology, Chongqing, China

**Keywords:** etomidate, propofol, electroencephalogram, consciousness monitors, neural network

## Abstract

**Objective:**

Establishing a convolutional neural network model for the recognition of characteristic raw electroencephalogram (EEG) signals is crucial for monitoring consciousness levels and guiding anesthetic drug administration.

**Methods:**

This trial was conducted from December 2023 to March 2024. A total of 40 surgery patients were randomly divided into either a propofol group (1% propofol injection, 10 mL: 100 mg) (P group) or a propofol-etomidate combination group (1% propofol injection, 10 mL: 100 mg, and 0.2% etomidate injection, 10 mL: 20 mg, mixed at a 2:1 volume ratio) (EP group). In the P group, target-controlled infusion (TCI) was employed for sedation induction, with an initial effect site concentration set at 5–6 μg/mL. The EP group received an intravenous push with a dosage of 0.2 mL/kg. Six consciousness-related EEG features were extracted from both groups and analyzed using four prediction models: support vector machine (SVM), Gaussian Naive Bayes (GNB), artificial neural network (ANN), and one-dimensional convolutional neural network (1D CNN). The performance of the models was evaluated based on accuracy, precision, recall, and F1-score.

**Results:**

The power spectral density (94%) and alpha/beta ratio (72%) demonstrated higher accuracy as indicators for assessing consciousness. The classification accuracy of the 1D CNN model for anesthesia-induced unconsciousness (97%) surpassed that of the SVM (83%), GNB (81%), and ANN (83%) models, with a significance level of *p* < 0.05. Furthermore, the mean and mean difference ± standard error of the primary power values for the EP and P groups during the induced period were as follows: delta (23.85 and 16.79, 7.055 ± 0.817, *p* < 0.001), theta (10.74 and 8.743, 1.995 ± 0.7045, *p* < 0.02), and total power (24.31 and 19.72, 4.588 ± 0.7107, *p* < 0.001).

**Conclusion:**

Large slow-wave oscillations, power spectral density, and the alpha/beta ratio are effective indicators of changes in consciousness during intravenous anesthesia with a propofol-etomidate combination. These indicators can aid anesthesiologists in evaluating the depth of anesthesia and adjusting dosages accordingly. The 1D CNN model, which incorporates consciousness-related EEG features, represents a promising tool for assessing the depth of anesthesia.

**Clinical Trial Registration:**

https://www.chictr.org.cn/index.html.

## 1 Introduction

General anesthesia is employed to induce a reversible loss of consciousness, followed by recovery. Variability in individual pharmacokinetics of intravenous anesthetics can lead to insufficient depth of anesthesia. Electroencephalogram (EEG) monitoring is extensively utilized for perioperative brain function assessment (1). Research (2) indicates that the combination of propofol and etomidate can mitigate intravenous pain, decrease the incidence of nausea, vomiting, and myoclonus, maintain hemodynamic stability during the induction of general anesthesia, and enhance cerebral oxygen metabolism. The loss of consciousness induced by the commonly used anesthetic drug propofol is characterized by an increase in low-frequency electroencephalogram (EEG) power (< 4 Hz) and the emergence of frontal alpha (8–12 Hz) oscillations (3, 4). However, the EEG characteristics of intravenous anesthesia with a propofol-etomidate combination are unknown, and changes in the state of consciousness after anaesthesia with this drug have not been reported.

Processed EEG indices (5–7), such as the Bispectral Index (BIS), Patient State Index (PSI), and Narcotrend, can monitor levels of consciousness. However, these indices may not accurately reflect the state of unconsciousness induced by all anesthetic drugs (8, 9). Consequently, tracking raw EEG changes has emerged as a research focus for anesthesiologists aiming to monitor consciousness levels (10, 11). To enhance the accuracy of anesthesia consciousness assessment, machine learning, and deep learning algorithms are increasingly employed in clinical EEG research (12–14). By extracting a large number of quantitative EEG features and combining them with machine learning, sedation levels can be predicted independently of the selected anesthetic drug (15). Among deep learning models, one-dimensional convolutional neural networks (1D CNNs) (16) are frequently utilized to process one-dimensional sequence data, including audio, text, and time series data such as electrocardiograms and electroencephalograms. These models are characterized by low computational requirements and are widely adopted in medical technology (17, 18). Therefore, employing 1D CNNs to identify and analyze features in raw EEG data is essential for enhancing the accuracy and convenience of assessing anesthesia awareness.

We constructed four models to assess the state of anesthesia consciousness using better-performing SVM, Gaussian Bayes (GNB), ANN, and self-developed 1D CNN architecture. Using EEG features relevant to distinguish between awake and sleeping states (19); i.e., the power spectral density (PSD), the fast and slow wave ratios (delta/beta, alpha/beta), the beta ratio, and phase-amplitude coupling (PAC) that allowed accurate tracking of changes in propofol anesthetic consciousness (3) and computationally efficient and artifact-resistant permutation entropy (20). The power spectral density shows the distribution of signal power per unit frequency range. A total of six EEG features were employed as the inputs of the neural network, with the output being a classification of three anesthesia stages: induction sedation, maintenance, and before extubation. In this study, induction sedation was defined as when the patient moved from wakefulness to a state of loss of consciousness, and the PSI value fell to 20–40 after intravenous infusion of propofol or propofol-etomidate combination. Anesthesia maintenance refers to the period between the infusion of intraoperative anesthesia drugs after tracheal intubation and the cessation of all anesthetic drug infusion. Before extubation refered to the period of time after stopping the anesthetic drug infusion, when the patient was resuscitated from anesthesia until he was awake for extubation. We compared the performance of our self-constructed 1D CNN model with the other three models and identified characteristic EEG signals that reflected changes in awareness induced by the two drugs. Furthermore, we utilized the 1D CNN to accurately classify anesthesia stages, thereby providing a mechanism for pharmaceutical robots to achieve targeted controlled anesthesia through EEG features.

## 2 Materials and methods

### 2.1 Study design and population

This study was registered with the Chinese Clinical Trial Registration Center (number: ChiCTR2300078715). All participants signed informed consent from December 2023 to March 2024. This trial enrolled 46 patients aged 18–40 years, who were scheduled to undergo maxillofacial surgery under general anesthesia, to participate in this prospective, observational, single-center study, in which the operation time was limited to 3 h. Subjects included in the study must have met the American Society of Anesthesiology (ASA) Class I physical condition, had a body mass index of 20–30 kg/m^2^, no long-term use of sedatives or psychotropic drugs, no history of alcohol abuse, and no stroke, epilepsy, brain damage or other brain complications.

### 2.2 Study procedures and data collection

The researchers employed computer-generated random numbers to randomly assign all subjects into two groups: a propofol group (1% propofol injection, 10 mL: 100 mg) (P group) and a propofol-etomidate group (1% propofol injection, 10 mL: 100 mg, and 0.2% etomidate injection, 10 mL: 20 mg, mixed at a 2:1 volume ratio) (EP group). eTable 1 summarizes the intravenous anesthesia regimen for both patient groups. During the operation, blood pressure was maintained within 20% of the baseline value. Sufentanil and cisatracurium were administered as appropriate by the anesthesiologist based on the procedure being performed. If intravenous anesthesia did not achieve the required depth of surgical anesthesia, sevoflurane inhalation anesthesia was introduced. All subjects were required to fast for 8 h before surgery. The EEG was continuously recorded using a Sedline Brain Function Monitor (Masimo Corporation) throughout the anesthesia process, from the time the patient entered the operating room until their exit. The monitoring electrodes included Fp1, Fp2, F7, F8, and the ground electrode Fpz, which were positioned to ensure an impedance of ≤5 kΩ, an amplitude of 5 μV/mm, a rolling speed of 30 mm/s, and a signal sampling rate of 179 Hz. The PSI (ranging from 0 to 100, where 100 indicates “fully awake” and 0 indicates “isoelectricity”), automatically generated by Sedline, served as an indicator of consciousness during anesthesia and provided an alternative to the BIS (21). The intraoperative depth of anesthesia was maintained at a PSI value between 25 and 50. Additionally, an electrocardiogram monitor was utilized to continuously track the patient’s vital signs, including non-invasive mean arterial pressure (MAP), heart rate (HR), electrocardiogram (ECG), respiratory rate, and peripheral blood oxygen saturation (SPO_2_). Intraoperative awareness was assessed using the modified Brice assessment (22).

### 2.3 Assessment of consciousness level

The Expert assessment of consciousness level (EACL) (23) represents the average anesthesia depth scores assigned by six experienced anesthesiologists, each possessing over 10 years of experience in the field. These scores were derived from both anesthesia records and the anesthesiologists’ professional judgment, establishing the EACL as a gold standard for assessing levels of consciousness. We used it as a reference standard for the output of machine learning models. In the context of general anesthesia, which encompasses induction, maintenance, and recovery phases, the depth of anesthesia transitions from shallow to deep and back to shallow. The EACL values obtained range from 0 to 100, akin to the Bispectral Index (BIS).

### 2.4 EEG preprocessing and feature extraction

The native EEG signal was resampled to 200 Hz, and EEG signals were preprocessed using MNE tool (24). A bandpass filter (0.5–40 Hz) as well as a Notch filter (50 Hz) were employed to remove baseline drift and wire noise interference. To further remove artifacts, researchers with experience in EEG recognition used a Python program written to graphically and manually cut EEG signals, discarding the highly intrusive portions and leaving clean EEG signals selectively. For each patient’s EEG, 2-min EEG segments were selected at each of the three anesthetic stages. Detailed information on EEG data was provided in eTable 2.

In this study, the PSD of the EEG signals was calculated using the Welch method, and the average absolute power of the signals in each frequency band [delta (0.5–5 Hz), theta (5–8 Hz), alpha (8–13 Hz), beta (13–26 Hz), and gamma (26–40 Hz)] were calculated according to the PSD. The YASA software library (25) was used to calculate the spectrogram features of the EEG signal with a sliding window of 2 s without overlap. A total of six EEG features were extracted in this study, where the beta ratio was defined as the ratio of the high-frequency band power (30–37 Hz) to the low-frequency band power (11–20 Hz), and permutation entropy was used as a typical nonlinear analytical method for measuring EEG signals during anesthesia and coma (20, 26). The PAC of delta to alpha was such that the phase of the low-frequency rhythmic delta modulated the amplitude of the high-frequency alpha wave oscillations, allowing for a more accurate assessment of arousal and anesthesia statuses. The modulation index (MI) was ideal for detecting PAC between two target frequency ranges. The detailed formulae for calculating the relevant values are shown in eAppendix 1.

### 2.5 Machine learning models and evaluation metrics

We selected four commonly used machine learning models: SVM (27), GNB (28), ANN (29), and our own constructed 1D CNN model. Neural networks have been extensively studied for EEG classification tasks (30–32). The first three models utilized the Scikit-learn machine learning library (33) for training and evaluation. Convolutional networks emphasize the extraction of local features and require less computational power, enabling them to learn EEG signals end-to-end from both the time and frequency domains (34). Gu et al. (32) argue that manual feature extraction is superior to directly inputting raw EEG data into the network for end-to-end learning. Consequently, we intentionally developed a more complex and effective 1D CNN model capable of accepting manually extracted EEG features. The proposed one-dimensional CNN model comprises five distinct types of layers: convolutional layers for feature extraction, pooling layers to reduce computational workload, batch normalization layers to stabilize model parameter learning, dropout layers to mitigate overfitting, and fully connected layers for classifying model outputs. In addition to the input layer, there are a total of four layers. The first three are feature extraction layers, all of which are one-dimensional convolutional layers, while the final output layer is a linear layer with three categories. For further details on the features and network structure, please refer to Table in eAppendix 1.

The deep learning framework used in the experiments was Pytorch (35). The common evaluation metrics of machine learning used in this study were accuracy, precision, recall, and the F1 score. Their formulas are detailed in eAppendix 1. We apply Gradient Weighted Class Activation Mapping (Grad-CAM) (36) to 1D CNNs to better account for the classification accuracy associated with the model features in Supplement 2.

### 2.6 Statistical methods

Previous relevant exploratory studies (32, 37) provided a sample size of approximately 20 cases and obtained stable dose estimates. This cohort study ultimately included 40 patients. Statistical analyses were performed using GraphPad Prism software version 10.0, and all data were subjected to the Shapiro–Wilk test to assess data normality, with normally distributed values expressed as the mean ± standard error or standard deviation, else as the interquartile range. Categorical variables are expressed as percentages. For basic information and anesthesia characteristics of univariate variables, an unpaired *t*-test was used to analyze normally distributed variables, the Mann–Whitney *U*-test for non-normal variables, and Fisher’s exact test for categorical variables. To analyze the impact of two anesthetic drugs on EEG characteristics and vital sign values at different stages of anesthesia, we conducted a two-factor analysis of variance (Two-way ANOVA), in which the comparison of the two types of drugs was between groups, and the comparison of each stage of anesthesia was within groups. The comparison between groups and within-group comparisons were performed using the Bonferroni correction and Tukey’s multiple comparisons test, respectively. The performance of the four model classification methods was compared using the non-parametric Wilcoxon signed-rank test. Statistical significance was considered to be achieved when the *p-*value was <0.05.

## 3 Results

### 3.1 Primary outcome

#### 3.1.1 Clinical characteristics of patients and machine learning

**Figure 1:**
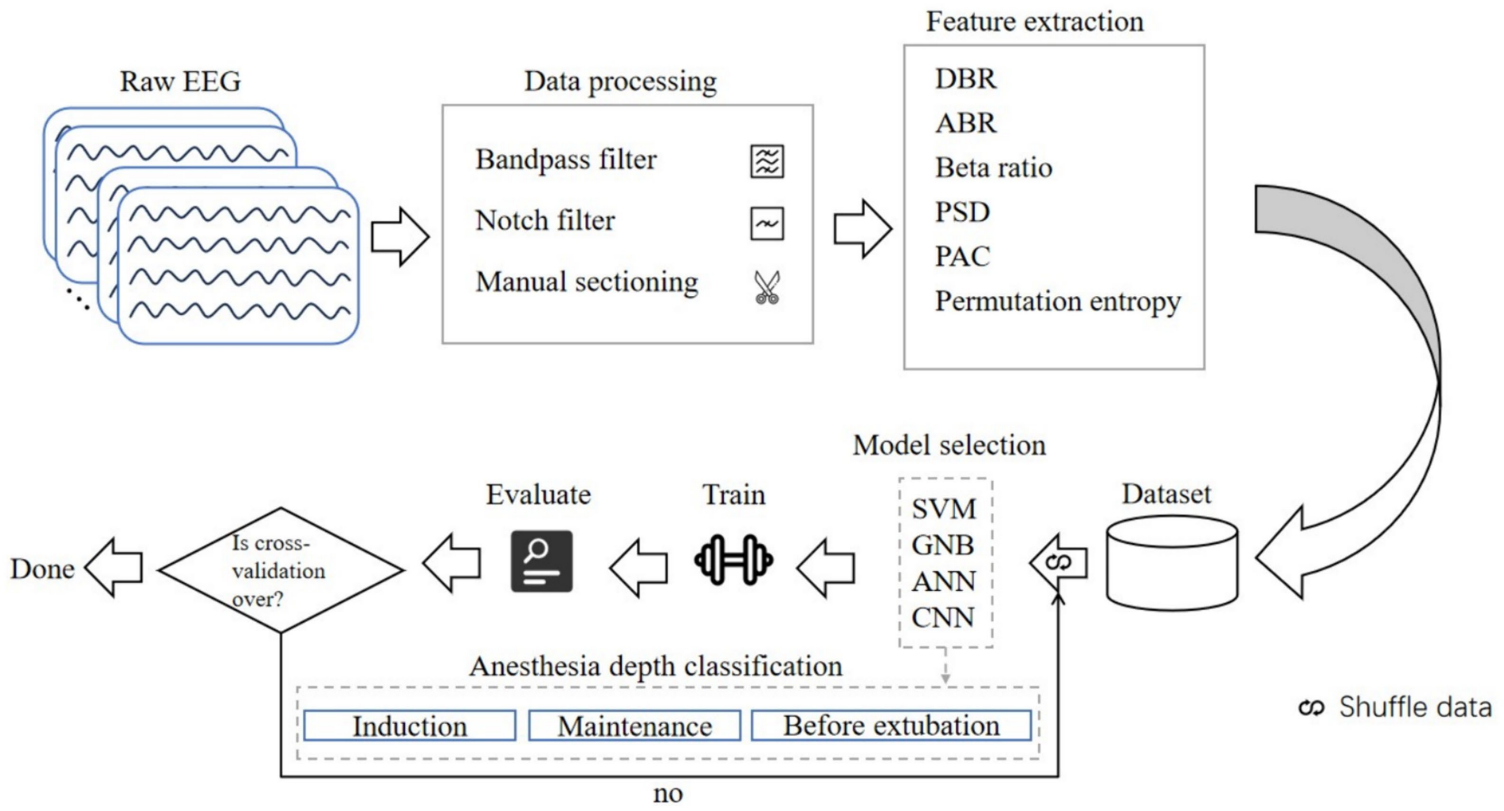
Flowchart of machine learning EEG features for classification of anesthesia awareness. EEG, electroencephalogram; DBR, delta/beta ratio; ABR, alpha/beta ratio; PSD, power spectral density; PAC, phase amplitude coupling; SVM, support vector machine; GNB, Gaussian Bayes; ANN, artificial neural network; CNN, convolutional neural network.

Four cases were excluded from the study, and there were two instances of intravenous anesthesia failure. In total, clinical characteristics from 40 young patients undergoing intravenous general anesthesia were analyzed (refer to eTable 3 and eFigure 1 for the grouping flowchart and clinical characteristics). All patients completed the entire anesthesia process, resulting in a sample size ratio of 40:40:40 across the three stages of anesthesia. We extracted 2-min artifact-free EEG segments for periodic analysis, yielding a total of 644 2-min EEG segments spanning the three stages of anesthesia. We calculated the EEG features using a sliding window of 2 s without overlapping. All feature information were flattened into one-dimensional features and stitched together. The ratio of the training set to the test set was established at 7:3, and 40-fold cross-validation was conducted on a random sample from the dataset. Figure 1 illustrates the flowchart of machine learning EEG features for classifying anesthesia awareness.

#### 3.1.2 Machine learning classification assessment

To identify significant EEG characteristic signals associated with consciousness, we individually combined each feature within the 1D CNN model, with the results presented in Table 1. The PSD showed higher accuracy (94%), followed by the alpha/beta ratio (72%), and the lowest accuracy was the PAC (53%). When all features were aggregated in the 1D CNN model, the classification accuracy increased to 97%. The inclusion of the PAC increased model accuracy from 94 to 97%, suggesting that the PAC was important for monitoring changes in levels of consciousness. Furthermore, we compared the performance of the 1D CNN models against the SVM, GNB, and ANN models, with the results detailed in Table 2. The precision, F1-score and classification accuracy of the CNN model across the three anesthesia stages surpassed those of the other three models. Statistical analyses indicated that the differences between the CNN and the other models were significant (*p* < 0.05).

**Table 1:** The results of 1D CNN learning single feature and multiple features.

Single feature	Classification accuracy	All features	Classification accuracy
Delta/Beta	58%	Include PAC diagram	97%
Alpha/Beta	72%	Not include PAC diagram	94%
Beta ratio (BR)	64%		
Permutation entropy	69%		
PSD	94%		
PAC diagram	53%		

PAC, Phase amplitude coupling; CNN, Convolutional neural networks; PSD, Power spectral density.

**Table 2:** Comparison of four models for anesthesia classification of EEG features.

	Precision induction	F1-score induction	Precision maintenance	F1-score maintenance	Precision before extubation	F1-score before extubation	Accuracy
CNN	100%	100%	100%	95%	94%	97%	97%
SVM	91%	80%	83%	91%	77%	80%	83%
GNB	90%	75%	77%	87%	77%	80%	81%
ANN	92%	85%	75%	82%	83%	83%	83%

CNN, Convolutional neural networks; SVM, Support vector machine; GNB, Gaussian naive Bayes; ANN, Artificial neural networks; EEG, electroencephalogram.

### 3.2 Exploratory outcomes

#### 3.2.1 Power spectrum analysis

**Figure 2:**
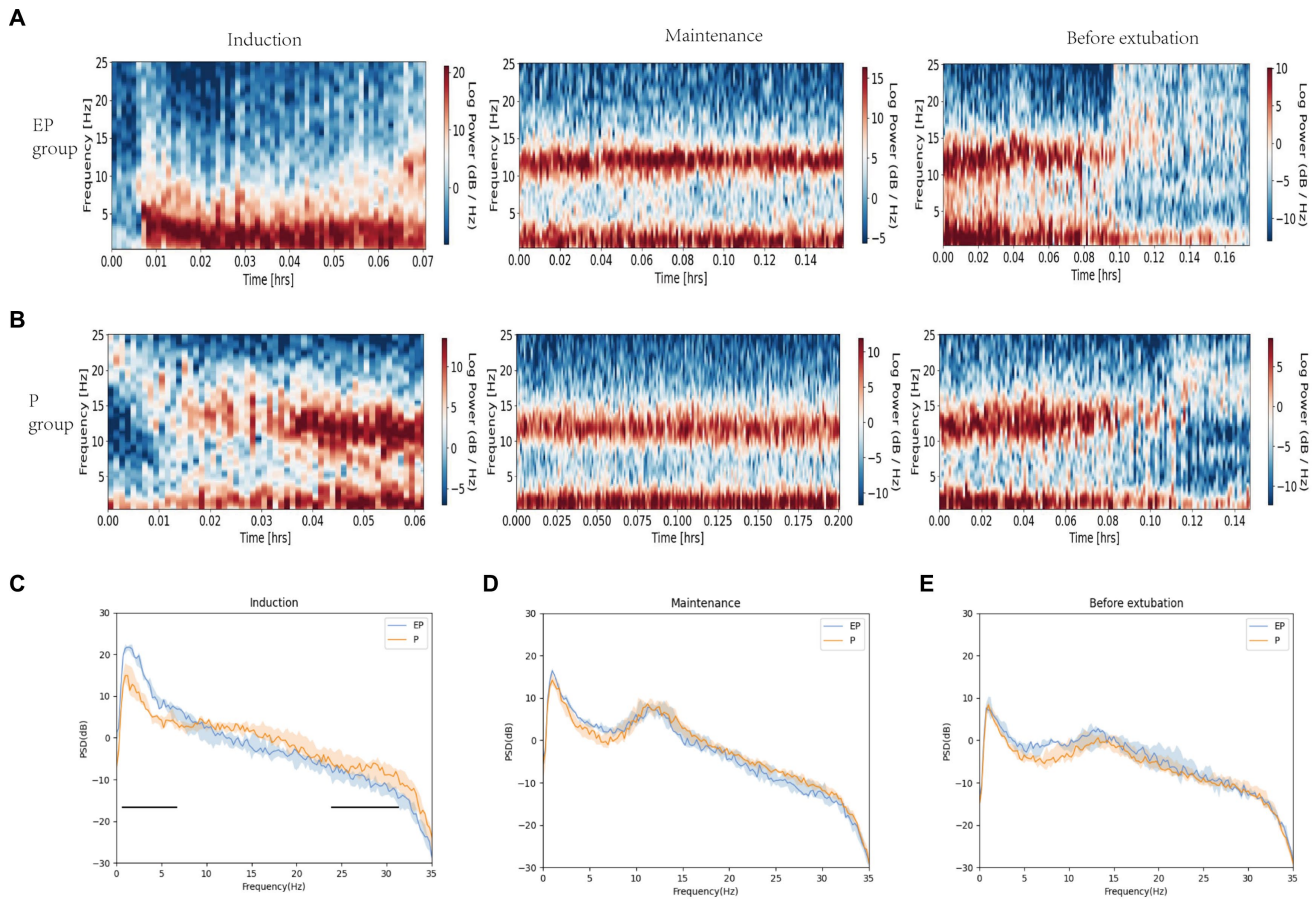
Spectrograms and spectral analysis of EP group and P group. **(A,B)** Spectrograms of EP group and P group in three stages; **(C–E)** Spectral analysis of EP group (Blue line, median; shaded area, 25th–75th percentiles) and P group (yellow line, median; shaded area, 25th–75th percentile) in three stages. During the induction period, the original EEG of the EP group showed alpha oscillations and significant data and theta oscillations, without obvious fast wave oscillations (13–40 Hz), while the original EEG of the P group showed beta, alpha, delta, and theta oscillations. The two black horizontal lines in **(C)** pointed out the spectral differences between the two groups of drugs in fast and slow wave oscillations. The data, theta, and total power values of both groups decreased after induction, while the alpha power value increased.

**Figure 3:**
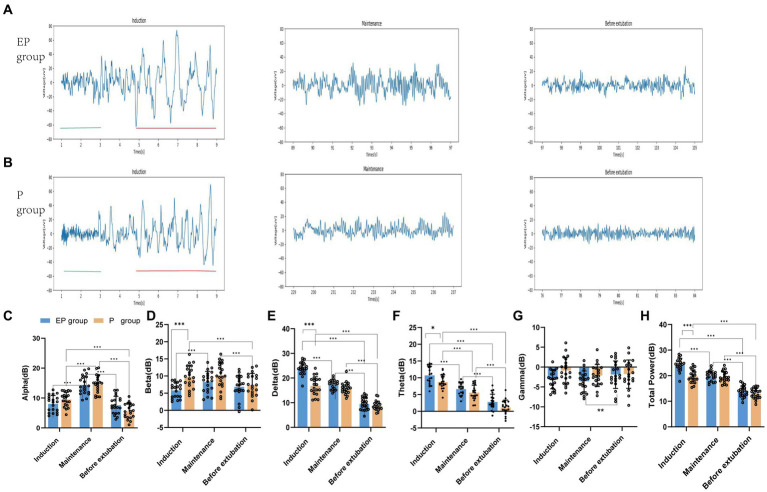
Raw EEG waveforms and five frequency band powers in the EP group and P group. **(A,B)** Raw EEG waveforms of the unprocessed Fp2 channel in the EP group and P group during the three stages of induction, maintenance, and before extubation. During the induction period, the distribution of red markers and green markers represent the difference between the two groups of slow-wave oscillations and fast-wave oscillations; **(C–H)** The power of each frequency band in the EP group and P group in the three stages vary within groups and between groups. Statistical significance is expressed as: *p < 0.033, **p < 0.002, ***p < 0.001.

The spectrograms (Figures 2A,B) and raw EEG waves (Figures 3A,B) of both groups exhibited similar oscillations in the delta, theta, and alpha bands during the maintenance period; both groups showed a “zip” opening pattern with beta-gamma oscillations during the pre-extubation period. Notably, the EP group displayed higher power in the delta and theta frequency ranges during the induction period in Figures 2C–E.

In Figures 3C–H, the statistical analysis of grouped histograms reveals significant differences. During the anesthesia induction and sedation period, the delta, theta and total power values of the EP group were notably higher than those of the P group. The between-group analysis indicated that, during the induction period, the means and mean differences ± standard errors for the power values of the EP group and the P group were as follows: delta (23.85 and 16.79, 7.055 ± 0.817, *p* < 0.001), theta (10.74 and 8.743, 1.995 ± 0.7045, *p* < 0.02), beta (5.842 and 9.825, −3.983 ± 1.008, *p* < 0.001), and total power values (24.31 and 19.72, 4.59 ± 0.711, *p* < 0.001), respectively. No significant differences were observed in the power values of the frequency bands between the two groups during the anesthetic maintenance period and the pre-extubation period. In the two-group analysis, the delta, theta, and total power values of the EP group were highest during the induction phase, followed by the maintenance phase, with the lowest values observed before extubation. The differences in delta power were 6.469 (induction vs. maintenance) and 7.835 (maintenance vs. before extubation). The differences in theta power were 4.081 (induction vs. maintenance) and 3.839 (maintenance vs. before extubation). Additionally, the differences in total power values were 4.395 (induction vs. maintenance) and 6.101 (maintenance vs. before extubation). The mean differences in alpha values were 6.191 (maintenance vs. induction) and 6.677 (maintenance vs. before extubation), with all *p*-values being <0.001. Similarly, the alpha and theta power values of the P group exhibited significant differences across the three phases. The differences in alpha power were 5.115 (maintenance vs. induction) and 3.907 (induction vs. before extubation), while the differences in theta power were 3.175 (induction vs. maintenance) and 4.401 (maintenance vs. before extubation), with all *p-*values also <0.001.

#### 3.2.2 PAC

**Figure 4:**
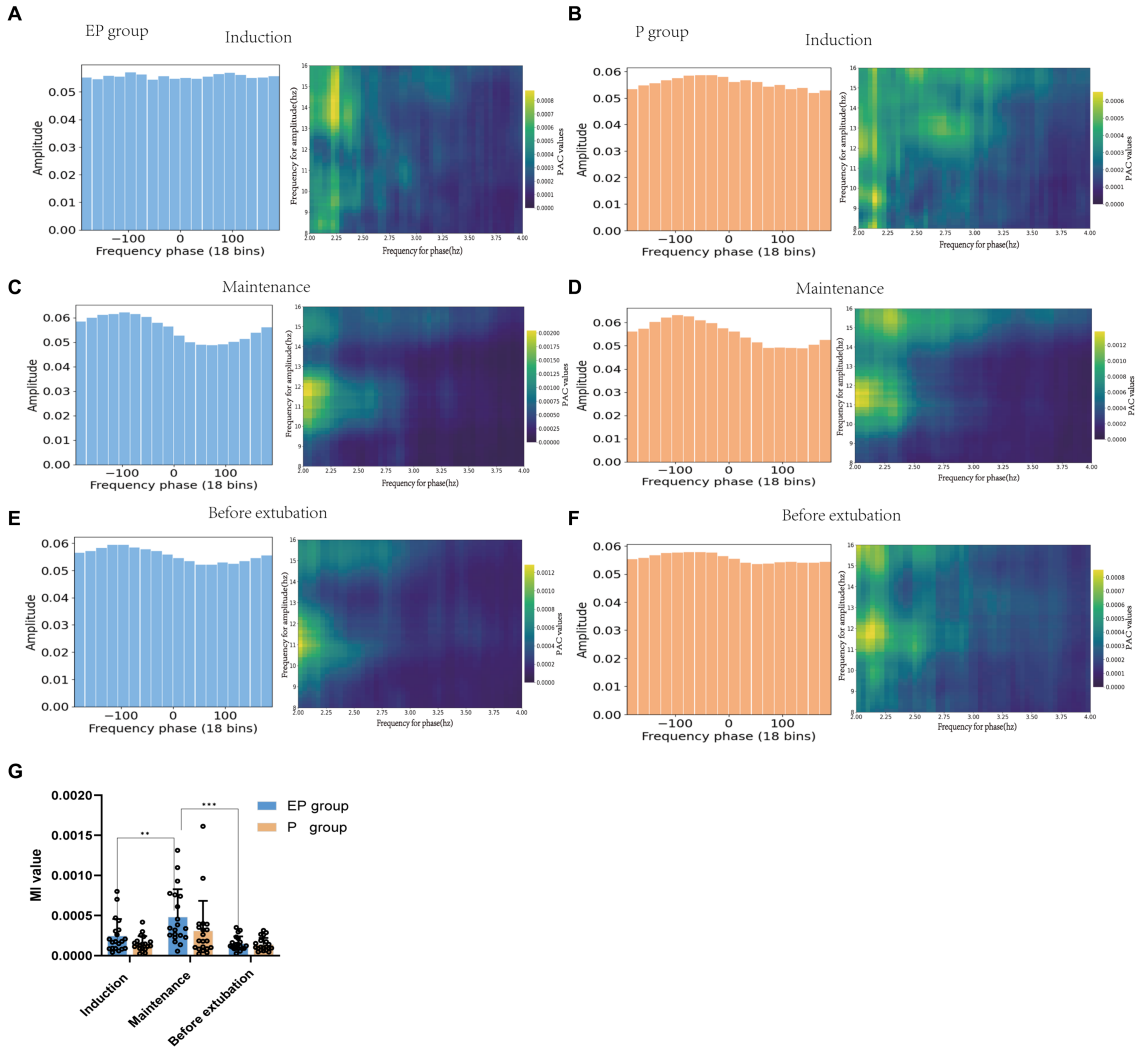
Phase-amplitude coupling patterns of patients in the EP group and P group. **(A,C,E)** represents the EP group. **(B,D,F)** represents the P group. The green picture on the right is the prefrontal cortex comodulograms, showing the modulation intensity of delta phase to high-frequency alpha band oscillation in various stages of anesthesia. Whether the average amplitude distribution of the phase-amplitude coupling diagram on the left is even or not represents whether the phase-amplitude coupling is missing; **(G)** The delta phase to alpha amplitude MI of patients in EP and P groups were compared during the three stages of anesthesia.

As illustrated in Figures 4A–F, the right comodulograms demonstrated that the modulation intensity of the delta phase on alpha oscillation in the EP and P groups was greater during the maintenance stage compared to the other two stages. Additionally, the left PAC graphs (Figures 4C,D) indicated that the average amplitude of the alpha band during the maintenance period was unevenly distributed within the delta phase [Statistical results: *p* < 0.001, test stationarity, Tensorpac tool (38)]. The coupling between the delta phase and alpha oscillation amplitude in both groups was further quantified using the MI value presented in Figure 4G. The statistical analysis revealed that the MI value for the EP group was notably higher during the maintenance period (maintenance vs. sedation, the mean difference was 0.0002, *p* < 0.05); however, no statistically significant differences were observed in MI values between the EP and P groups. Therefore, alpha-delta PAC was found to be stronger in both the EP and P groups during the anesthesia maintenance period.

## 4 Discussion

This study employed deep learning techniques to categorize anesthesia-induced unconsciousness based on frontal electroencephalogram features in young patients receiving intravenous anesthesia with either propofol or a propofol-etomidate combination. Six types of EEG signals, associated with two drug groups and different states of consciousness, were extracted for model input. The developed 1D CNN model was compared against SVM, GNB, and ANN regarding their accuracy in classifying the three anesthesia states. All four models effectively identified the three anesthesia states, with the CNN model exhibiting the highest accuracy. The various EEG signals, as well as the alpha/beta ratios and power spectrum features, exhibited high accuracy. These findings suggest that the power spectrum and alpha/beta ratio can serve as straightforward and interpretable methods for predicting patient consciousness during anesthesia in clinical practice. Our 1D CNN model shows promising advantages in predicting the consciousness state during propofol intravenous anesthesia. Additionally, the study revealed marked differences in EEG patterns between the two groups during induced sedation. The P group initially displayed prominent beta and alpha oscillations, transitioning to delta and theta oscillations. In contrast, the EP group exhibited substantial high-amplitude delta and theta oscillations, along with reduced alpha oscillations, and higher total power. Interestingly, EEG patterns in the EP group resembled those of the P group during the maintenance and before extubation periods.

Our research demonstrates that various EEG features associated with consciousness, including frequency domain features, entropy features, and phase-amplitude coupling features, serve as inputs to our model. By categorizing three stages of anesthesia—induction, maintenance, and before extubation—as outputs, our CNN model achieves superior accuracy in classifying anesthesia states compared to traditional machine learning methods. We employed a tailored 4-layer 1D CNN architecture designed to decode band power characteristics (39). Previous studies (40, 41) have utilized CNNs for classification learning. The computational demand is minimal, as the convolutional layer effectively extracts local features while the pooling layer reduces data dimensionality. We implemented Dropout layers, BatchNorm layers, and the Adamw (42) optimization method, along with 40-fold cross-validation, to effectively mitigate model overfitting. Additionally, our Grad-CAM technique provided further evidence of the 1D CNNs model’s proficiency in recognizing input features.

Compared to previous studies employing machine learning to monitor states of consciousness, our model performed well. Two recent studies (15, 43) conducted machine learning on EEG signals of healthy volunteers under sedation with multiple anesthetic drugs. These models, which selected multiple quantitative EEG features to track propofol-induced unconsciousness, achieved an average area under the curve (AUC) exceeding 0.95. However, these studies focused solely on sedation and did not encompass deep anesthesia. In contrast, our study involved the classification of conscious states during anesthesia throughout surgical procedures. Furthermore, an evaluation of consciousness state classification based on four types of EEG features combined with ANN and SVM models (32) revealed relatively low classification accuracy for propofol intravenous anesthesia (ANN: 79.1%, SVM: 76.7%), thereby underscoring the superiority of our model. Dubost et al. (19) identified that the frontal lobe channel F8 and the temporal lobe channel T7 are optimal for detecting anesthesia depth, with the prefrontal cortex serving as a crucial node in the arousal circuit (44). The selection of 10 spectral features that effectively distinguish between awake and sleeping states better predicted anesthesia depth. Our study’s results further indicate that PSD derived from frontal lobe EEG monitoring, utilized as input for the 1D CNN model, can more accurately classify anesthesia consciousness. Additionally, the accuracy is expected to improve further by integrating various consciousness-related EEG signals.

The EEG features of propofol-etomidate and propofol administration are associated with gamma-aminobutyric acid type A (GABAA) receptors in the central nervous system. The sedative effect of propofol is related to the GABAA receptor subtype and the position of the β1, β2, and β3 subunits of the transmembrane domain (45). Etomidate regulates and activates the β2 and β3 subunits of the transmembrane domain by acting on the GABAA receptor, while exerting minimal influence on the β1-containing receptor (46). The distinct binding sites for etomidate and propofol at the GABA receptor elucidate the basis for their differing affinities. Therefore, propofol-etomidate operates similarly to propofol (47, 48), as both agents inhibit neuronal firing in the cortex, thalamus, and reticular formation (49), and induce highly structured thalamocortical oscillations alongside slow oscillations that contribute to fragmentation of cortical activity (3, 4). Our experiments preliminarily show that this large-amplitude slow oscillation mode can be used as the EEG characteristic of propofol-etomidate administration resulting in loss of consciousness under general anesthesia. Additionally, we observed quantitative differences in the EEG power spectra between the two drugs. This observation aligns with Lei Zhang’s experimental findings (50), which suggest that the neural circuit mechanisms underlying etomidate-induced loss of consciousness are closely associated with the enhancement of coherence in delta, alpha, and theta waves, resulting in an increased total power spectrum value. A study (49) comparing intravenous anesthesia using propofol alone versus etomidate found that both drugs induced peak EEG power in the 12 to 13 Hz range. However, etomidate anesthesia also exhibited oscillations in the 7 to 8 Hz band, indicating more pronounced EEG changes compared to propofol. Our study results suggest that etomidate may alter EEG characteristics following propofol anesthesia, particularly with high doses administered over a short duration. Specifically, the combination of high doses of propofol and etomidate during the induction phase of anesthesia resulted in a low-frequency, high-amplitude EEG pattern. In contrast, the dose of etomidate during the maintenance and recovery phases did not significantly impact propofol-induced EEG characteristics. Future research could explore the specific etomidate dosage or concentration that could affect propofol’s EEG signal. These drug-induced EEG patterns associated with consciousness, such as delta waves and power spectral density, were utilized as model features to enhance model performance.

Studies have identified delta oscillations, spectral slope changes, and increases in alpha power associated with propofol anesthesia (51–53) as potential biomarkers for loss of consciousness. Specifically, Purdon et al. (3) found that GABA-type general anesthetics modulate the PAC of the alpha amplitude through a low-frequency phase, disrupting thalamocortical neuron transmission and thereby inhibiting the spread of information within the brain. This mechanism is also implicated in the loss of consciousness. Our study demonstrated that both treatment groups maintained a robust coupling of the delta phase to the alpha amplitude following intravenous anesthesia, with the anesthesia maintenance phase exhibiting significantly stronger coupling than both the induction and recovery phases. This finding may serve as an indicator for assessing the depth of anesthesia. The spectrogram and PAC observed in our study align with previous research findings (54). The mechanism of the effect of the two drugs on the PAC of neural activity in human cortical and subcortical regions remains to be unequivocally elucidated (54). Helfrich et al. (55) found that weakened or decoupled PAC is related to brain atrophy and cognitive function impairment. Therefore, PAC can not only track the state of anesthesia but also reflect the degree of brain health, providing valuable insights for predicting the impact of anesthetic drugs on cognitive impairment. Although PAC demonstrated a relatively low classification accuracy (53%) in this study’s model, its inclusion among multiple EEG feature inputs enhanced the model’s classification accuracy from 94 to 97%.

Our experimental approach had several limitations. Firstly, the training sample size was relatively small, and the test population comprised young and healthy patients undergoing oral and maxillofacial surgery. Consequently, our findings cannot be generalized to other age groups, individuals with frail health, or different types of surgeries; thus, larger and more diverse cohort studies are necessary. Secondly, the applicability of our 1D CNN model to other types of anesthetic drugs necessitates further validation. Furthermore, although we enhanced the interpretability of the model by optimizing feature extraction, streamlining the model structure, utilizing appropriate evaluation performance metrics, and employing heat maps generated by Grad-CAM, additional clinical test sets and other interpretability tools are required for further validation.

## 5 Conclusion

In summary, the machine learning model based on a 1D CNN can more effectively leverage original EEG signals for automated analysis, positioning it as an innovative tool for evaluating anesthesia depth. Notably, the observation of significant slow-wave oscillations, changes in power spectral density, and variations in the alpha/beta ratio can provide anesthesiologists with a straightforward and practical method to assess alterations in consciousness levels induced by intravenous anesthetic agents.

## Data Availability

The raw data supporting the conclusions of this article will be made available by the authors, without undue reservation.
