# Supplementary material for: Unconscious classification of quantitative electroencephalogram features from propofol versus propofol combined with etomidate anesthesia using one-dimensional convolutional neural network
Source: Front Med (Lausanne). 2024 Sep 18;11:1447951. doi: 10.3389/fmed.2024.1447951 (PMC11445052; doi:10.3389/fmed.2024.1447951)
Supplement: Supplementary file 1 [file Data_Sheet_1.docx]

**Supplemental Content**

**eTable 1：** Intravenous anesthesia scheme for EP group and P group

**eTable 2：** The information of the EEG data.

**eAppendix 1:** EEG feature computation methods, convolutional neural network models and supplementary clinical information

**eReferences**

**eFigure 1:** Grouping flow chart of two drugs.

**eTable 3:**  Patients clinical characteristics.

**eFigure 2:**  Important vital sign changes in the two drugs anesthesia .

**eTable 1 Intravenous anesthesia scheme for EP group and P group**

| Groups | EP group | P group |
| --- | --- | --- |
| Induction sedation | EP mixture 0.2ml/kg,IV(slowly) | Transvenous target controlled infusion (TCI)(Schneider pharmacokinetic model):Propofol 5-6ug/ml |
| After induction sedation | Sufentanil 0.4ug/kg ,IV;  cisatracurium besylate 0.15mg/kg,IV | |
| Maintenance | EP mixture 0.5-0.7ml/(kg.h) ,IV;  remifentanil 0.1-0.2ug/(kg.min) ,IV | Propofol 4-12mg/(kg.h), IV;  remifentanil 0.1-0.2ug/(kg.min),IV |

(EP: propofol-etomidate combination,P:propofol.)

（Detailed anaesthesia protocol：in P group, propofol sedation was induced via a multichannel infusion workstation (HP-30pro; Medcaptain MEDICAL Technology Co., Ltd.; Shenzhen, China) via an intravenous line to guide target-controlled infusion (TCI), and the infusion mode was selected (Schneider pharmacokinetic model, maximum flow rate <700 mL/h) was used to predict the effect site concentration, and the initial effect site concentration (Ce) of propofol was 5-6ug/ml.The sedation induction dose of 0.2 ml/kg was selected in the EP group by mixing 1% propofol injection (10 ml: 100 mg) and 0.2% etomidate injection (10 ml: 20 mg) in a volume ratio of 2:1.When the patient's consciousness disappeared and the PSI value was 25-30, intravenous push sufentanil 0.4ug/kg and cisatracurium benzenesulfonate 0.15mg/kg were given, and after the anaesthesia drugs took effect, tracheal intubation and mechanical ventilation were performed. Intraoperative anaesthesia was maintained with remifentanil at a maintenance dose of 0.1-0.2ug/(kg.min), propofol at 4-12mg/(kg.h) continuously pumped intravenously in P group, and the propofol-etomidate combination at 0.5-0.7 ml/(kg.h) continuously pumped intravenously in EP group, to maintain the PSI value between 25-50.）

**eTable 2 The information of the EEG data.**

| Subject | EEG duration during anesthesia induction（x 2min） | EEG duration during anesthesia maintenance（x 2min） | EEG duration during anesthesia recovery（x 2min） |
| --- | --- | --- | --- |
| Patient 1 | 2 | 5 | 3 |
| Patient 2 | 1 | 9 | 1 |
| Patient 3 | 2 | 4 | 1 |
| Patient 4 | 2.5 | 12.5 | 3 |
| Patient 5 | 1 | 18 | 3 |
| Patient 6 | 1.5 | 3.5 | 5 |
| Patient 7 | 2 | 20 | 6.5 |
| Patient 8 | 2 | 8 | 9 |
| Patient 9 | 1 | 16 | 3.5 |
| Patient 10 | 1.5 | 9.5 | 8 |
| Patient 11 | 1.5 | 11.5 | 4 |
| Patient 12 | 1.5 | 12 | 5.5 |
| Patient 13 | 1 | 15.5 | 1.5 |
| Patient 14 | 1.5 | 13 | 1.5 |
| Patient 15 | 2 | 6 | 2 |
| Patient 16 | 2 | 11 | 3 |
| Patient 17 | 1 | 12.5 | 4 |
| Patient 18 | 1.5 | 7.5 | 2.5 |
| Patient 19 | 1.5 | 7 | 2 |
| Patient 20 | 1.5 | 28 | 1 |
| Patient 21 | 1 | 2.5 | 2.5 |
| Patient 22 | 1 | 17.5 | 2 |
| Patient 23 | 1.5 | 13 | 2 |
| Patient 24 | 2 | 1 | 1 |
| Patient 25 | 1.5 | 21 | 1 |
| Patient 26 | 2.5 | 6 | 3 |
| Patient 27 | 2 | 8.5 | 3 |
| Patient 28 | 1.5 | 21 | 3 |
| Patient 29 | 1 | 18.5 | 1 |
| Patient 30 | 2.5 | 4.5 | 13 |
| Patient 31 | 1.5 | 9 | 1 |
| Patient 32 | 1.5 | 10 | 4 |
| Patient 33 | 2 | 10 | 7 |
| Patient 34 | 1.5 | 10 | 2.5 |
| Patient 35 | 2.5 | 7 | 3 |
| Patient 36 | 1.5 | 6 | 6 |
| Patient 37 | 2 | 11 | 3.5 |
| Patient 38 | 1 | 7.5 | 1.5 |
| Patient 39 | 2 | 17 | 4.5 |
| Patient 40 | 1.5 | 10 | 4 |
| Add up | 65 | 440.5 | 138.5 |
| Total | 644 | | |

**eAppendix 1 EEG feature computation methods, convolutional neural network models and supplementary clinical information**

（1）The amplitude and frequency structure of the EEG signal were initially described by the average power of the traditional frequency bands: delta (0.5–4 Hz), theta (5–7 Hz), alpha (8–14 Hz), and beta (15 –25 Hz).The power in each frequency band was then logarithmically transformed so that all spectral powers were expressed in dB units (10 × base 10 logarithm, referenced to 1 µV 2 /Hz). The calculation formula for the ratio of Delta power to Beta power, the ratio of alpha power to Beta power and the Beta ratio can be expressed as:

$$DBR= log(\frac{bandpower(5-8hz)}{bandpower(13-26hz)})$$

$$ABR= log(\frac{bandpower(8-13hz)}{bandpower(13-26hz)})$$

$$Beta ratio= log(\frac{bandpower(30-37hz)}{bandpower(11-20hz)})$$

（2）Permutation entropy ^1^ It is a quantitative complexity analysis of dynamic time series.Given a time series of N points,$X=\{x\left( 1 \right),x\left( 2 \right),\ldots,x(N)\}$,vector $X\left( i \right)=\{x\left( i \right),x\left( i+L \right),\ldots,x(i+(m-1)L)\}$,$1\leq i\leq N-(m-1)$, where $m$ is the embedding dimension,and $L$ is the time delay.The interior of each $X\left( i \right)$ can be rearranged in increasing order, and the arranged vector $X\left( i \right)$ can be represented as $\left\{ x\left( i+\left( j_{1}-1 \right)L \right),x\left( i+\left( j_{2}-1 \right)L \right),\ldots,x\left( i+\left( j_{m}-1 \right)L \right) \right\}$.The subscript order $J=\left\{ j_{1},j_{2},\ldots,j_{m} \right\}$ is called permutation.The M-dimensional vector $J$ has $K=m!$possible permutations.$P_{c}$ represents the probability of the permutation occurring.The normalized permutation entropy can be expressed as:

$$PE=\frac{-\sum_{c=1}^{K} P_{c}*\ln P_{c}}{\ln K}$$

（3） Phase amplitude coupling Before calculating PAC, it was necessary to analyze the stationarity of the EEG signal. This study first used 2-4 Hz and 8-16 Hz band-pass filters to filter the time series, and then used the Hilbert transform to obtain the corresponding phase time series
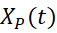
 and the amplitude time series
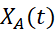
. For each phase/amplitude pair, the phase (range [ -π to π]) was divided into 18 bins. Each $X_{A}\left( t \right)$ is assigned to equally spaced phase bins according to the value of $X_{P}(t)$.This study first calculates the average amplitude value of all values in each bin and expresses it as $A_{m}\left( i \right),(i=1,2,\ldots,18)$, Then the average amplitude value is divided by the average amplitude value of all bins and normalized to obtain the phase amplitude plots,expressed as：$M\left( i \right)=A_{m}(i)/\sum_{i=1}^{18} A_{m}(i)$.The modulation index (MI) is calculated by Kullback-Leibler distance,and the formula is$MI=\sum_{i=1}^{18} {M\left( i \right)*\log\left( M\left( i \right) \right)}/{\log\left( 18 \right)}+1$.When the MI value is 0, there is a lack of phase-amplitude coupling. The greater the MI value is, the greater the phase amplitude coupling degree is, and the amplitude distribution is uneven. The tool used to calculate PAC in this experiment was Tensorpac ^2^. By constructing comodulograms, it was possible to assess how the amplitude of the high-frequency signal was modulated by the phase of the low-frequency oscillations ^3^. The comodulograms were constructed for both frequency band intervals using high-resolution sampling of 50 samples.

（4）This study's neural network was designed as one-dimensional convolutional neural network (1D CNN) where the number of channels increased with the number of convolutions, while the signal sequence length decreased with pooling. Its structure has a total of 4 layers.The first 3 layers are all 1-dimensional convolution layers as feature extraction layers.The input layer is not used as a layer in the network. It accepts extracted features rather than direct raw EEG signals. The last layer is a 3-category linear layer as the output layer, followed by a softmax function to obtain the probability of each output category. The network uses 1-dimensional maxpooling with a kernel size of 2 for feature pooling, which reduces the feature length by half. The neural network uses the BatchNorm layer to stabilize the parameter learning of the model to prevent gradient explosion and gradient disappearance. The network structure is shown in **Table**. Each row includes the name of the network layer and its specific parameters. In the **Table**, C represents the output feature length of the last convolutional layer in the network, which varies with the number of features used in the input data. The activation function used in the network is the Gelu function. We used a Dropout layer, and the dropout rate was chosen to be 0.5 to prevent the model from overfitting.The loss function chosen is CrossEntropyLoss, which can be expressed as:

$$Loss=-\sum_{n=1}^{N} y_{n}*\log\hat{y}_{n}$$

Where $y_{n}$ is the true value and $\hat{y}_{n}$ is the predicted value.The learning rate $l$ is set to ${10}^{-4}$, and the training batch size is 32. The optimization method selected is the AdamW method^4^.This method uses weight decay in parameter update to achieve the same results as L2 regularization to prevent model overfitting.The optimization algorithm calculates the gradient of the network parameters and updates the network parameters in order to minimize the loss function.

The configuration of this experimental environment is as follows:the graphics card is NVIDIA GeForce RTX 3060 Ti, the processor is AMD Ryzen 5 5600 6-Core Processor, the system memory is 32G, and the deep learning framework is Pytorch.

**Table** Convolutional neural network structure

| Layer name | Parameters |
| --- | --- |
| Conv1d | In_channels=1,out_channels=64,kernel_size=1 |
| BatchNorm1d | In_channels=64 |
| Conv1d | In_channels=64,out_channels=128,kernel_size=1 |
| BatchNorm1d | In_channels=64 |
| MaxPool1d | Kernel_size=2 |
| Conv1d | In_channels=128,out_channels=256,kernel_size=1 |
| BatchNorm1d | In_channels=128 |
| MaxPool1d | Kernel_size=2 |
| Linear | In_features=C,out_channels=3 |

（5） In terms of model training, we selected 2 min of EEG data from the manually extracted clips during each of the three anesthesia periods and calculated the EEG features using a sliding window of 2 s without overlapping. All feature information were flattened into one-dimensional features stitched together. The training cycle was 300 trials and the model parameters with the best evaluation results were saved throughout the training process. The ratio of training to validation sets was 7:3, and a 40-fold cross-validation was performed with random samples in the dataset. The entire dataset had a total of 120 samples and the ratio of each class was 40:40:40.

（6） In this study, common evaluation metrics for machine learning are used: accuracy, precision, recall, and F1-score. Here TP stands for true positive, TN stands for true negative, FP stands for false positive, and FN stands for false negative.

The accuracy rate refers to the proportion of all correct predictions to all prediction results, which can be expressed as:

$$Accuracy=\frac{TP+TN}{TP+TN+FP+FN}$$

The precision rate is expressed as the proportion of correctly predicted positives to predicted positive results,which can be expressed as:

$$Precision=\frac{TP}{TP+FP}$$

The recall rate is expressed as the proportion of correctly predicted positive results to the true positive results, which can be expressed as:

$$Recall=\frac{TP}{TP+FN}$$

The precision rate and recall rate affect each other and restrict each other. F1-score can balance the advantages of two points, and it is expressed as

$$F1=2*\frac{(Precision*Recall)}{Precision+Recall}=\frac{2TP}{2TP+FP+FN}$$

（7）Supplementary clinical information

Our study has found that propofol-etomidate could maintain intravenous anesthesia for brief surgery, with stable respiratory and circulation systems without obvious adverse effects. The results were roughly the same as those reported in previous studies on propofol, etomidate and their combination^5-8^. In our study, intravenous induction of anesthesia by propofol was an infusion method controlled by target concentration in the effect chamber, which can maintain better hemodynamic stability than manually controlled infusion of propofol ^9^. The incidence of injection pain and myoclonus during anesthesia induction in the present research was higher than reported in previous studies. In order to better reflect the EEG changes of the two drugs, our study did not administer midazolam, lidocaine, opioids or dexmedetomidine in advance to reduce discomfort ^10,11^. In addition, the combined drugs were administered at a volume ratio of 2:1, which reduced the dosage of etomidate, the intensity of myoclonus and the incidence of nausea and vomiting caused by etomidate. There was no significant decrease in oxygen saturation in this study due to pre-oxygenation before anesthesia which ensured a high oxygen reserve.

The anesthesia-induced sedation dose of the propofol-etomidate combination in this study was based on the results of previous studies^8,12^. Since there are few reports of this combination being used for intravenous anesthesia maintenance, our study was guided by the PSI values and changes in vital signs, and the anesthesia maintenance dose for the EP group of the present study was determined to be 0.5-0.7 ml/(kg. h). More studies are needed to explore the intravenous anesthesia maintenance dose of propofol-etomidate combination.

During general anesthesia, it was essential to document vital signs (including HR, blood pressure, and SPO2), anesthetic events (such as the administration of induction medications, loss of consciousness, endotracheal intubation, intraoperative anesthesia maintenance, recovery of consciousness, and extubation), surgical events (including the initiation and conclusion of surgery and occurrences of significant stimulation), as well as other clinical manifestations (such as tearing, sweating, abnormal body movements, and signs of arousal).

**eReferences**

1. Cao Y, Tung W-w, Gao J, Protopopescu VA, Hively LMJPrE. Detecting dynamical changes in time series using the permutation entropy. *Physical review E.* 2004;70(4):046217.

2. Combrisson E, Nest T, Brovelli A, et al. Tensorpac: An open-source Python toolbox for tensor-based phase-amplitude coupling measurement in electrophysiological brain signals. *PLoS Comput Biol.* 2020;16(10):e1008302.

3. de Hemptinne C, Swann NC, Ostrem JL, et al. Therapeutic deep brain stimulation reduces cortical phase-amplitude coupling in Parkinson's disease. *Nat Neurosci.* 2015;18(5):779-786.

4. Loshchilov I, Hutter FJapa. Decoupled weight decay regularization. *arXiv preprint arXiv.* 2017;1711:05101.

5. Chen L, Liang X, Tan X, Wen H, Jiang J, Li Y. Safety and efficacy of combined use of propofol and etomidate for sedation during gastroscopy: Systematic review and meta-analysis. *Medicine (Baltimore).* 2019;98(20):e15712.

6. Meng QT, Cao C, Liu HM, et al. Safety and efficacy of etomidate and propofol anesthesia in elderly patients undergoing gastroscopy: A double-blind randomized clinical study. *Exp Ther Med.* 2016;12(3):1515-1524.

7. Rathore VS, Singh S, Taank P, Khandelwal A, Kaushal A. Clinical Analysis of Propofol, Etomidate and an Admixture of Etomidate and Propofol for Induction of General Anaesthesia. *Turkish journal of anaesthesiology and reanimation.* 2019;47(5):382-386.

8. Yağan Ö, Taş N, Küçük A, Hancı V, Yurtlu BS. Haemodynamic Responses to Tracheal Intubation Using Propofol, Etomidate and Etomidate-Propofol Combination in Anaesthesia Induction. *J Cardiovasc Thorac Res.* 2015;7(4):134-140.

9. Passot S, Servin F, Allary R, et al. Target-controlled versus manually-controlled infusion of propofol for direct laryngoscopy and bronchoscopy. *Anesthesia & Analgesia.* 2002;94(5):1212-1216.

10. Doenicke AW, Roizen MF, Kugler J, Kroll H, Foss J, Ostwald PJTJotASoA. Reducing myoclonus after etomidate. *Anesthesiology.* 1999;90(1):113-119.

11. Honarmand A, Safavi MJCdi. Prevention of propofol-induced injection pain by sufentanil: a placebo-controlled comparison with remifentanil. *Clin Drug Investig.* 2008;28:27-35.

12. Du J, Bie X, Zhu D. Application of etomidate and propofol mixture in hematoma removal in patients with intracranial epidural hematoma. *American journal of translational research.* 2021;13(7):8403-8408.

**eFigure 1:** **Grouping flow chart of two drugs.**


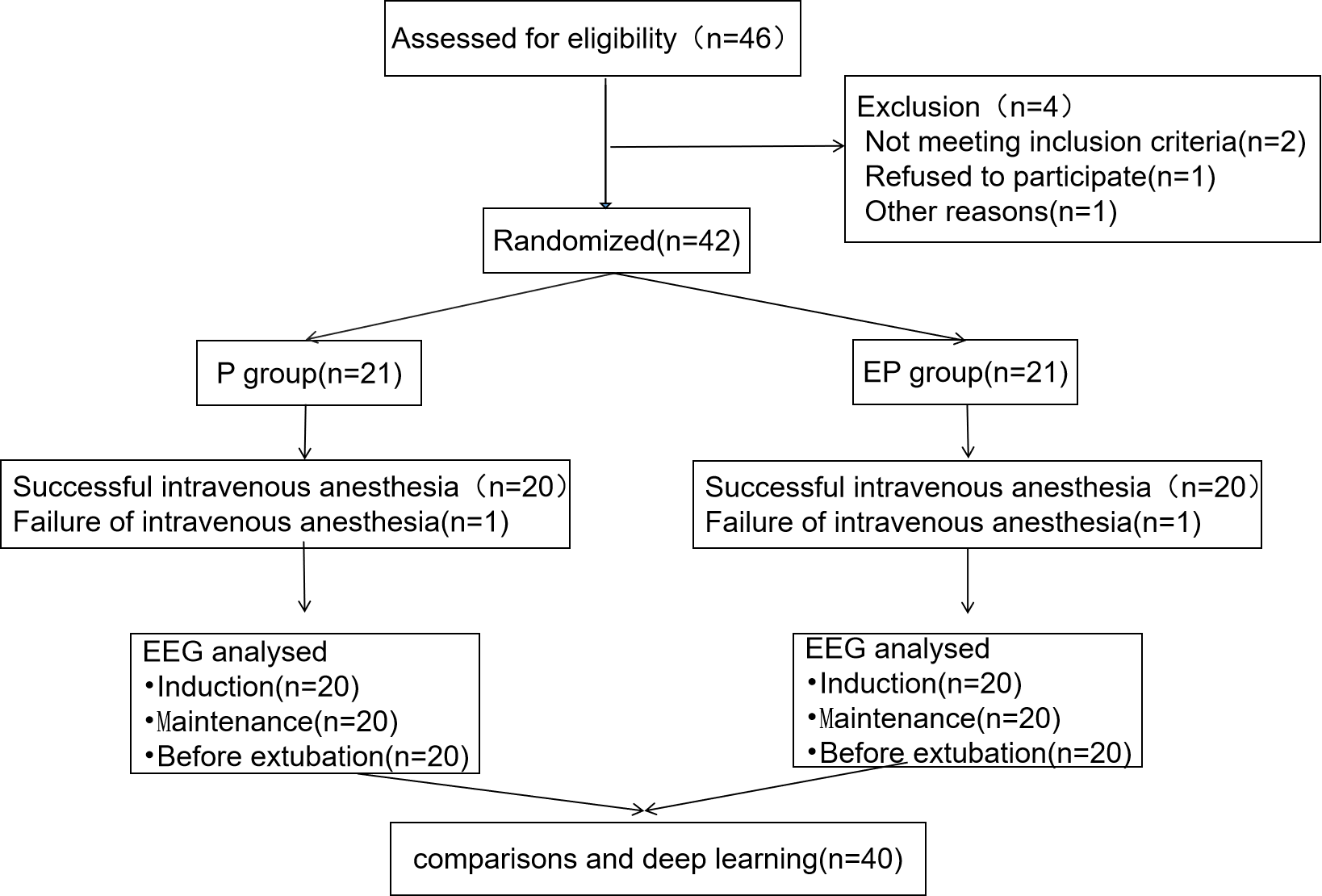


(P, propofol injection; EP, Propofol and etomidate combination; EEG, electroencephalogram)

**eTable 3 Patients clinical characteristics**

| **Variable** | **EP Group**  **（n=20）** | **P Group**  **（n=20）** | ***P* value** |
| --- | --- | --- | --- |
| Age(range)(years) | 30(19-38) | 29(20-39) | 0.5388 |
| BMI(Kg/m2) | 21.6±3.36 | 22.2±2.96 | 0.6101 |
| Male/female | 11/9 | 11/9 | >0.9999 |
| Length of surgery(min) | 50(36-89) | 48(31-75) | 0.5864 |
| Length of anesthesia(min) | 81(57-118) | 65.5(57-95) | 0.6731 |
| PSI value during tube extraction | 85.5±3.93 | 84.6± 4.74 | 0.4943 |
| Extubation time(min) | 7(3-12) | 7.5(6-10.8) | 0.5680 |
| SPO2 value after sedation(%) | 99(97-99) | 99(98-99) | 0.9908 |

(EP: propofol and etomidate mixture,P: propofol,PSI: patient status index.Values are indicated by the mean(±SD).Differences were considered significant if *P*<0.05.)

**eFigure 2 Important vital sign changes in the two drugs anesthesia .**


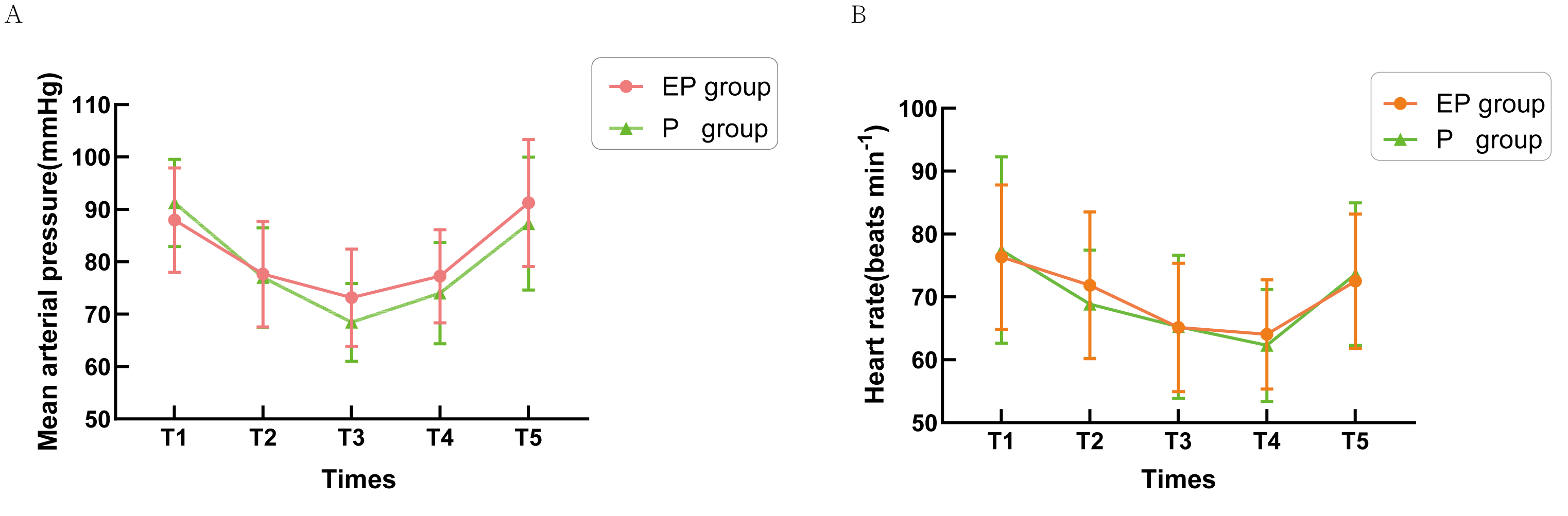


(A and B show the five stages of vital signs of two anesthetic drugs when awake (T1), 5 minutes after sedation induction (T2), 5 minutes after anesthesia induction (T3), 5 minutes after the start of surgery (T4), and 2 minutes after recovery from anesthesia (T5). It can be seen that the changes in heart rate and mean arterial pressure of the two groups during each stage of anesthesia are consistent.)
