# Supplementary material for: Unconscious classification of quantitative electroencephalogram features from propofol versus propofol combined with etomidate anesthesia using one-dimensional convolutional neural network
Source: Front Med (Lausanne). 2024 Sep 18;11:1447951. doi: 10.3389/fmed.2024.1447951 (PMC11445052; doi:10.3389/fmed.2024.1447951)
Supplement: Supplementary file 2 [file Data_Sheet_2.docx]

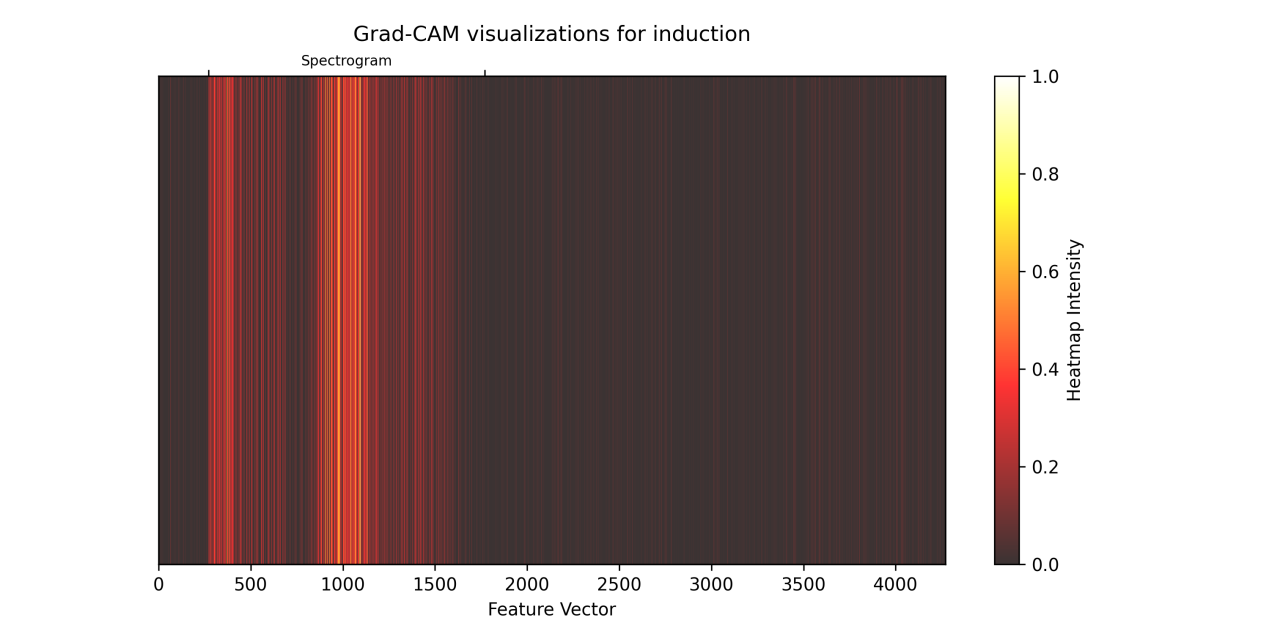

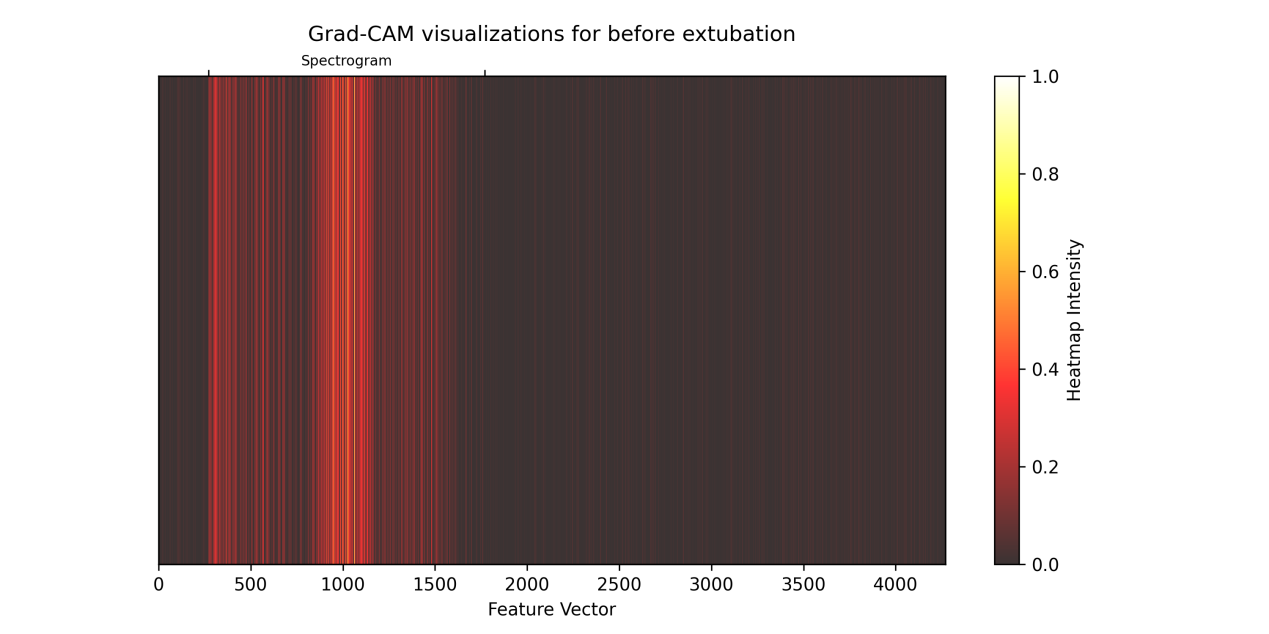

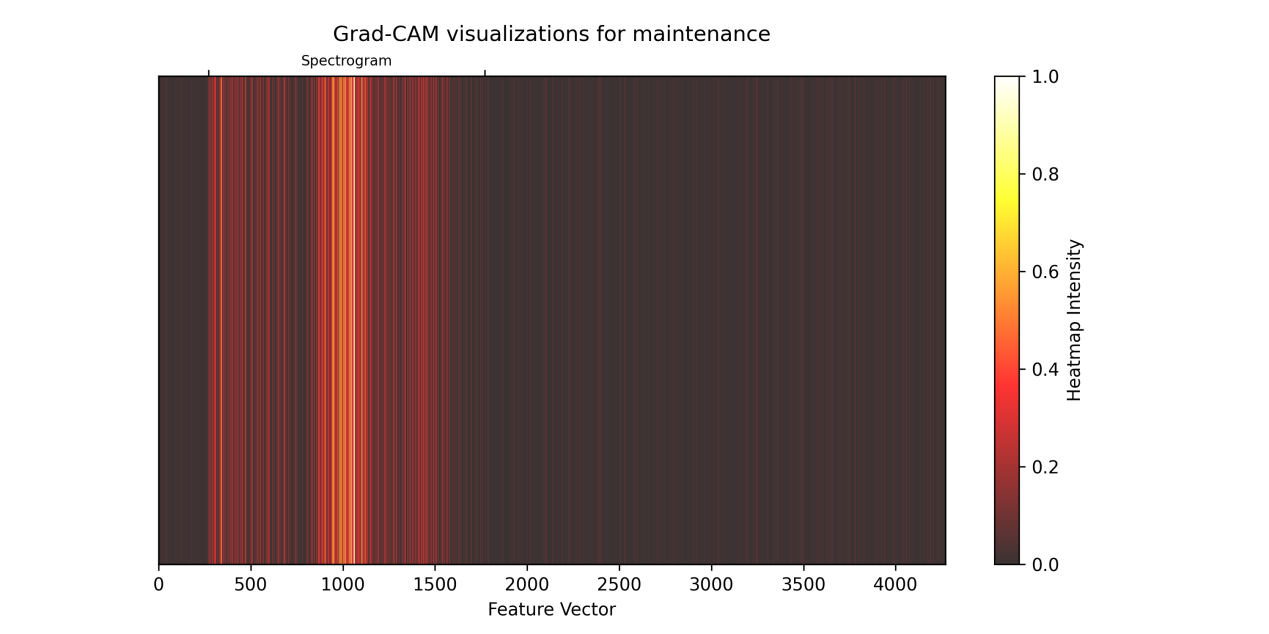


Gradient-weighted Class Activation Mapping (Grad-CAM)[1] is a technique to increase the interpretability and transparency of convolutional neural networks. It is widely used in the field of computer vision to provide intuitive interpretability in a visual way. Porting it to our task, we do not display the heatmap superimposed on the input data because our input data is extracted EEG features. The y-axis of the graph is aesthetically meaningless, the x-axis represents a one-dimensional vector, and the thermal intensity is normalised to values from 0 to 1. X ranges from 270 to 1770 representing the feature of power spectrogram. In the figure, it can be seen that the heat values in this range are high in all the three classifications, indicating that the feature has a great influence on the model in making classification decisions for the final output.

1. Selvaraju R R, Cogswell M, Das A, et al. Grad-cam: Visual explanations from deep networks via gradient-based localization[C]//Proceedings of the IEEE international conference on computer vision. 2017: 618-626.
